# Investigating iron intake in risk of progression from islet autoimmunity to type 1 diabetes: The diabetes autoimmunity study in the young

**DOI:** 10.3389/fimmu.2023.1124370

**Published:** 2023-03-28

**Authors:** Sulafa Elhassan, Fran Dong, Teresa Buckner, Randi K. Johnson, Jennifer A. Seifert, Patrick M. Carry, Lauren Vanderlinden, Kathleen Waugh, Marian Rewers, Jill M. Norris

**Affiliations:** ^1^ Department of Epidemiology, Colorado School of Public Health, University of Colorado Anschutz Medical Campus, Aurora, CO, United States; ^2^ Barbara Davis Center for Diabetes, School of Medicine, University of Colorado Anschutz Medical Campus, Aurora, CO, United States; ^3^ Department of Kinesiology, Nutrition, and Dietetics, University of Northern Colorado, Greeley, CO, United States; ^4^ Department of Biomedical Informatics, School of Medicine, University of Colorado Anschutz Medical Campus, Aurora, CO, United States; ^5^ Department of Medicine, Division of Rheumatology, School of Medicine, University of Colorado Anschutz Medical Campus, Aurora, CO, United States; ^6^ Colorado Program for Musculoskeletal Research, Department of Orthopedics, University of Colorado Anschutz Medical Campus, Aurora, CO, United States

**Keywords:** type 1 diabetes, autoimmunity, iron intake, diet, children

## Abstract

**Background:**

Studies of the role of iron in the risk of type 1 diabetes (T1D) have been inconsistent. Given that iron generates reactive oxygen radicals, which can lead to oxidative damage and apoptosis in the beta cells of the pancreas, we examined whether iron intake was associated with the risk of progressing to T1D in individuals with islet autoimmunity (IA), the pre-clinical phase of T1D.

**Methods:**

DAISY is a prospective cohort following 2,547 children at increased risk for IA and progression to T1D. IA is defined as at least two consecutive serum samples positive for at least one autoantibody (insulin, GAD, IA-2, or ZnT8). We measured dietary intake at the time of IA seroconversion in 175 children with IA, and of these, 64 progressed to T1D. We used Cox regression to examine the association between energy-adjusted iron intake and progression to T1D, adjusting for HLA-DR3/4 genotype, race/ethnicity, age at seroconversion, presence of multiple autoantibodies at seroconversion, and multiple vitamin use. In addition, we tested whether this association was modified by vitamin C or calcium intake.

**Results:**

In children with IA, high iron intake (as defined as above the 75th percentile, > 20.3 mg/day) was associated with decreased risk of progression to T1D compared to moderate iron intake (as defined by the middle 25-75th percentiles, 12.7-20.3 mg/day) (adjusted hazard ratio (HR): 0.35; 95% confidence interval (CI): 0.15, 0.79). The association between iron intake and T1D was not modified by vitamin C nor calcium intake. In a sensitivity analysis, the removal of six children who had been diagnosed with celiac disease prior to IA seroconversion did not affect this association.

**Conclusion:**

Higher iron intake at the time of IA seroconversion is associated with a lower risk of progression to T1D, independent of multivitamin supplement use. Further research that includes plasma biomarkers of iron status is needed to investigate the relationship between iron and the risk of T1D.

## 1 Introduction

Type 1 diabetes (T1D) is a chronic disease characterized by the destruction of insulin-producing cells in the pancreas. The increasing incidence of T1D in children (1) and the significant morbidity associated with its complications highlight the necessity of identifying potential ways in which to prevent or delay the progression of the disease.

T1D is characterized by a pre-clinical phase defined by the presence of autoantibodies against pancreas cell antigens, termed islet autoimmunity (IA). Variation in the risk and timing of progression to T1D in individuals with IA (2–5), and variation in metabolomic (6) and transcriptomic (7) signatures of progressors suggest that environmental and/or genetic factors may play a role in whether or not one develops T1D during this time. We have shown that higher glycemic index (8), total sugars intake (9), and higher cow milk protein/lactose intake (10) were associated with a higher risk of progression to T1D in children with IA. Moreover, a dietary pattern characterized by higher total sugars intake and lower intake of B vitamins was associated with an increased risk of progression to T1D (11).

Diabetes has been linked to an excess of iron, given that iron produces reactive oxygen species (ROS), which can cause beta cells in the pancreas to undergo oxidative damage and cell death (12). While free iron is necessary for regular cell activity and glucose homeostasis, it may be damaging in excess. Although iron supplementation during pregnancy was associated with increased T1D risk in the offspring in a Norwegian birth cohort (13), no associations were observed in Danish (14) or Finnish (15) cohort studies. A small case-control study reported that T1D cases had a higher iron intake in the first four months of life compared with controls (16), while a larger Danish cohort observed that intake of iron supplements during the first 18 months of life was associated with lower risk of T1D (14). The inconsistencies between these studies may be due to the fact that dietary habits can affect iron absorption and bioavailability and may modify the relationship between iron and risk of T1D.

Higher vitamin C intake is an effective enhancer of non-heme iron absorption due to its reducing and chelating characteristics (17). On the other hand, calcium intake is associated with a decreased absorption of both heme and non-heme iron (17). Inconsistent associations suggest that more research is needed to determine the complex relationship between dietary iron intake and the risk of T1D.

We examined whether iron consumption is associated with the risk of progression to T1D in children with islet autoantibodies and whether this association is modified by vitamin C or calcium intake.

## 2 Methods

From 1993-2004, DAISY recruited 2,547 children in Colorado who were at high risk for developing T1D (18). Prospective follow-up for the development of islet autoantibodies and T1D included clinic visits at 9, 15, and 24 months, and annually thereafter (19). An IA case is defined by the presence of one or more confirmed autoantibody to insulin, GAD65, IA-2, or ZnT8 on at least two consecutive clinic visits. Age at seroconversion is defined as the age of the child at the first visit at which IA was detected. IA cases follow an accelerated protocol with clinic visits every 3-6 months until T1D is diagnosed by a physician following standard criteria, including typical symptoms of polyuria, polydipsia and/or weight loss and a random glucose , > 11.1 mmol/l or an OGTT with a fasting plasma glucose ≥ 7.0 mmol/l or 2-h glucose ≥ 11.1 mmol/l (20). The Colorado Multiple Institutional Review Board approved all DAISY study protocols (COMIRB 92-080). Informed consent was obtained from the parents/legal guardians of all children. Assent was obtained from children aged 7 years and older. All research was performed in accordance with relevant guidelines/regulations.

### 2.1 Dietary intake assessment

Dietary intake was measured annually using the Willett semi-quantitative food frequency questionnaire (FFQ). Starting when the child was two years old, parents completed an FFQ representing their child’s average intake over the previous year until age 10 years, after which study participants self-reported using the Youth Adolescent Questionnaire (YAQ). The FFQ has been previously validated (21), and the FFQ and YAQ shown to be comparable in the DAISY study population (22).

The average amounts of daily nutrient intakes were estimated from the FFQ and YAQ at the Channing Laboratory, Harvard, MA. Nutrient variables included the intake from both foods and supplements, unless otherwise noted. The residual method was used to calculate the energy-adjusted nutrient intakes (23). For each nutrient, residuals from the linear regression of total calories on nutrient intake are saved and scaled by adding the population mean nutrient intake as a constant.

Our primary exposure variable is iron intake (from food and supplements), which we analyzed as both a continuous and a categorical variable. We categorized iron intake as high (>75th percentile), moderate (25th-75th percentiles), and low (< 25th percentile), and used the moderate category as the reference group. We examined energy-adjusted vitamin C intake and calcium intake (from food and supplements) as effect modifiers. Both vitamin C and calcium were treated as continuous variables.

Multiple vitamin users were defined as individuals reporting intake of more than one vitamin or mineral supplement. 71% of multiple vitamin users were consuming supplemental iron, either as part of a multivitamin or as a single supplement.

### 2.2 Statistical analyses

During DAISY follow-up, 219 children developed IA. Children with IA that were missing FFQ data at their seroconversion visit (n=42) or whose seroconversion visit occurred less than 3 months prior to T1D diagnosis (n=2) were excluded from all analyses. Of the 175 children with IA in the study cohort, 64 developed T1D.

We used Cox regression models to examine the association between energy-adjusted iron intake at seroconversion (categorized as high, moderate, and low) and time to progression to T1D adjusting for HLA-DR3/4 genotype, race/ethnicity (non-Hispanic white (NHW) vs other), age at seroconversion visit (in years), multiple antibodies at seroconversion visit (> 1 vs 1 autoantibody) and multiple vitamin use (yes/no). All variables met the proportional hazards assumption based on the supremum test. We included interaction terms to test whether vitamin C or calcium intake modified the association between iron intake and progression to T1D; the significance of the interaction terms was evaluated by the -2 log-likelihood test for improvement of model fit. Data analyses were performed with SAS v9.4 (SAS Institute Inc., Cary, NC). Two-tailed p-values <0.05 were considered significant.

## 3 Result

**Table 1** provides descriptive data on demographics and dietary intake variables of the 175 IA-positive children by whether they progressed to T1D. Children who progressed to T1D were more likely to be non-Hispanic white, have the high-risk HLA genotype, an earlier age at seroconversion visit, and had multiple positive autoantibodies at the seroconversion visit compared to those who did not. Moreover, children who progressed to T1D were less likely to be in the high iron intake category than in the moderate intake category (**Table 1**).

**Table 1:** Descriptive and dietary intake characteristics of children with IA with unadjusted hazard ratios (HR) for progression to T1D.

	Progressed to T1D (n=64)	Did not progress to T1D (n=111)	Unadjusted HR ( 95% CI), p-value
Descriptive Characteristics			
	Mean (SD)	Mean (SD)	
Age at seroconversion (years)	4.9 (3.8)	8.7 (4.9)	0.89 (0.82, 0.97), p = 0.0053
Time to T1D/last follow-up (years)	6.6 (3.9)	10.9 (5.5)	**…………**
	n (column %)	n (column %)	
Sex (Female)	30 (46.9)	56 (50.5)	0.89 (0.55, 1.45), p =0.6445
Race-ethnicity (NHW)	58 (90.6)	80 (72.1)	2.73 (1.18, 6.32), p = 0.0193
HLA DR3/4 (Positive)	26 (40.6)	30 (27.0)	1.94 (1.17, 3.23), p = 0.0105
First-degree relative with T1D (yes)	41 (64.1)	66 (59.5)	1.11 (0.67, 1.85), p = 0.6904
Multiple positive autoantibodies at seroconversion visit (yes)	25 (39.1)	15 (13.5)	2.14 (1.32, 3.48), p = 0.0021
Body Mass Index [weight(kg)/height(m)^2^]	16.8 (2.7)	17.6 (3.3)	0.98 (0.87,1.09), p = 0.6656
Dietary Intake Characteristics			
	Mean (SD)	Mean (SD)	
^*^Iron intake (mg/day)	16.1 (6.6)	18.3 (7.7)	0.73 (0.54, 1.01), p = 0.0540
^*^Iron from food only (mg/day)	13.4 (3.0)	14.6 (4.3)	0.80 (0.62, 1.02), p = 0.0731
^*^Vitamin C intake (mg/day)	190.7 (135.5)	168.8 (155.7)	1.03 (0.84, 1.26), p = 0.8078
^*^Calcium intake (mg/day)	1307.9 (453.3)	1264.1 (421.8)	1.07 (0.86,1.34), p = 0.5258
	n (column %)	n (column %)	
^*^Iron intake Categories			
High (>20.3 mg/day)	8 (12.5)	36 (32.4)	0.33 (0.15, 0.70), p = 0.0041
Moderate (12.7-20.3mg/day)	38 (59.4)	51 (46.0)	1.0 (reference)
Low (<12.7 mg/day)	18 (28.1)	24 (21.6)	0.87 (0.50, 1.50), p = 0.6154
Multiple vitamin use (yes)	29 (45.3)	58 (52.3)	0.65 (0.40, 1.06), p = 0.0819

*Energy-adjusted variables.

Our adjusted analysis suggests a significant association between high iron intake and decreased risk of progression to T1D compared with moderate iron intake (HR: 0.31; 95% CI: 0.14, 0.72; P-value: 0.0065) (**Table 2**). As the iron association is opposite to that of our *a priori* hypothesis, we explored a potential alternate hypothesis that high intake of iron is representing another factor. We observed that children who were categorized into the high iron intake category (>75th percentile) were more likely to be taking multiple vitamin supplements, where 39 (88%) of children in the high iron category took multiple vitamins compared with 34 (38%) of children in the moderate iron category, and 14 (33%) of those in the low iron category, suggesting that the inverse association between iron and progression to T1D may be confounded by multiple vitamin supplement use. However, after adjusting for multiple vitamin use, the association between high iron intake and lower risk of progression to T1D remained significant (HR: 0.35; 95% CI: 0.15, 0.79; P-value: 0.0116). Moreover, the interactions of iron intake with vitamin C and calcium intake on risk of progression to T1D were not statistically significant (-2 LL p-values: 0.61 and 0.41, respectively), and thus were not included in the final model.

**Table 2:** Adjusted hazard ratios of iron intake categories and progression to T1D among 175 children with IA.

	Without Adjustment for Multiple Vitamin Use		With Adjustment for Multiple Vitamin Use	
	Adjusted HR (95% CI)	P-value	Adjusted HR (95% CI)	P-value
*Iron Intake Category				
High	0.31 (0.14, 0.72)	0.0065	0.35 (0.15, 0.79)	0.0116
Moderate	1.0 (reference)		1.0 (reference)	
Low	0.61 (0.35, 1.07)	0.0864	0.60 (0.33, 1.08)	0.0883
Multiple Vitamin Use (Yes)	–	–	0.80 (0.49, 1.32)	0.3906
Age at Seroconversion visit (years)	0.89 (0.82, 0.91)	0.0086	0.89 (0.82, 0.97)	0.0073
Multiple positive autoantibodies at seroconversion visit (Yes)	1.44 (0.84, 2.46)	0.1889	1.44 (0.84, 2.48)	0.1843
Race/ethnicity (NHW)	2.46 (1.08, 5.61)	0.0330	2.63 (1.12, 6.21)	0.0269
HLA DR3/4 (Positive)	2.28 (1.35, 3.86)	0.0022	2.21 (1.30, 3.77)	0.0036

*Energy-adjusted variable.

Given that the presence of celiac disease may affect the absorption of dietary iron, we conducted a sensitivity analysis in which we removed from our dataset six children who had been diagnosed with celiac disease prior to IA seroconversion. The removal of the children with celiac disease did not change the findings with regard to iron intake and progression to T1D (data not shown), suggesting that the presence of celiac disease was not affecting the results.

## 4 Discussion

High iron intake was associated with a 65% decreased risk of progressing from IA to T1D compared with moderate iron intake, independent of multivitamin use. This observation does not support our *a priori* hypothesis that high iron intake from diet and supplements would increase the risk of progression to T1D; however, it is consistent with the observation from a large Danish cohort that intake of iron supplements during the first 18 months of life was associated with lower risk of developing T1D (14). Our study extended the Danish birth cohort study by showing that iron intake was associated with lower risk of T1D by reducing the risk of progression from autoantibody positivity to T1D.

Iron is an important mineral for children, as it is essential for many cellular functions, including erythrocyte function and oxygen transport, brain development, and immune system support (24). Moreover, iron intake prevents the development of anemia in children, which can cause fatigue and decrease physical activity. According to the National Institutes of Health (NIH) the recommended daily allowance (RDA) for iron consumption through food and supplements ranges from 13.7-16.3 mg per day in children and young adults (2-19 years) (25), which is equivalent to the “moderate” category in our study. Children in our “high” category had intake just above the RDA levels. In the United States, iron intake (from food alone) is 11.5–13.7 mg for children aged 2–11 years and 15.1 mg for children ages 12–19 years (26). The inter-quartile range of iron intake from foods was 11.8-15.4 mg, indicating that the DAISY children were not consuming a large amount of iron. In fact, the DAISY children who did consume “high” and “moderate” amounts of iron were eating quantities comparable to children in the U.S (26). Due to this, we may not have been able to test the hypothesis that very high levels of iron, i.e., ‘overload’, was associated with progression to T1D in this study

The role of iron in the etiology of T1D is likely complex. We have recently observed gene expression changes during IA that differed between those that progressed to T1D, those that reverted to an autoantibody negative state, and those that remained autoantibody positive (7). Our enrichment and co-expression analyses identified a biological pathway with the hub gene, lactoferrin (LTF), which encodes an iron-binding milk protein involved in the regulation of iron homeostasis (27). Lactoferrin is able to strongly and reversibly bind iron ions, allowing the body to maintain iron homeostasis to prevent iron toxicity. Moreover, studies in the NOD mouse suggest that T1D risk may be associated with a potential defect in pancreatic iron homeostasis (28).

We had hypothesized that the association between iron intake and T1D progression would be modified by vitamin C intake because vitamin C increases the absorption of iron. The fact that we did not find this may be due to low power or perhaps a more complex relationship between the two nutrients, given that iron is involved in the free radical synthesis and vitamin C is one of the chemical compounds that functions as a chelator and can influence the pace of the free radical production. Therefore, despite the fact that vitamin C can increase iron in the blood, it also may help to decrease the impact of iron on T1D (29). The relation between iron and immunity is strong because anti-inflammatory macrophages are greatly responsible for body iron distribution and consequently shift to a more proinflammatory state in iron-deficient conditions (30, 31). High iron levels lead to increased hepcidin production, which inhibits further iron accumulation in macrophage and liver cells and decreases dietary iron absorption, resulting in a decrease in serum iron (31). However, when more iron is required, hepcidin levels drop, allowing macrophages to release iron and enhancing the intestinal ability to absorb dietary iron (31). This also explains why chronic immune activation causes anemia of inflammation, in which dietary iron absorption is decreased and is not improved by vitamin C intake (32).

We had also hypothesized that the association between iron intake and T1D progression would be modified by calcium intake because calcium decreases the absorption of iron. While the lack of detectable interaction may be due to low power, an alternative explanation is that the effect of calcium on iron absorption might only last a short while, and adaptation might happen over time (33).

As noted previously, T1D incidence has been increasing 3% per year since 1960 (1). Our observation that increased iron intake is associated with decreased risk of progression to T1D may offer one explanation for this increasing incidence, since dietary intake of iron has decreased in 1-17 year old between 1999-2015 in the U.S. National Health and Examination Survey (NHANES) (34).

A strength of this study is that it is a prospective cohort study that followed the children with IA closely, with measurement of dietary intake prior to the development of the T1D outcome. We acknowledge that our dietary data are self-reported, which may result in measurement error in estimating the amount of iron, vitamin C and calcium in the children’s diet. However, this type of error would likely bias our association towards the null, thus suggesting that our hazard ratio may be an underestimate of the effect of iron. Moreover, our results apply to children who are at increased risk for T1D with islet autoimmunity; it is unclear whether our results can be generalized to the general population.

It is possible that our observation that high iron intake was associated with a decreased risk of progression to T1D might be due to the healthier lifestyle habits of children reporting higher intakes of iron. Future studies using iron status biomarkers, such as serum ferritin, will be helpful in investigating iron as a risk factor for the progression of T1D and any unknown aspects of this relationship.

In conclusion, higher iron intake was associated with a lower risk of developing T1D in children with IA compared with moderate intake. However, further studies should be conducted to determine unknown factors that can explain the inverse association between iron and the risk of T1D before recommendations can be made due to the potential for iron toxicity.

## Data availability statement

The data analyzed in this study is subject to the following licenses/restrictions: The datasets generated during and/or analyzed during the current study are available from the corresponding author upon reasonable request. Requests to access these datasets should be directed to jill.norris@cuanschutz.edu.

## Ethics statement

The studies involving human participants were reviewed and approved by University of Colorado. Written informed consent to participate in this study was provided by the participants’ legal guardian/next of kin.

## Author contributions

SE and JN led the study design, statistical analyses, statistical interpretation of data, drafted the manuscript, and approved the final version. FD, TB, RJ, JS, PC, LV, KW and MR substantially contributed to the acquisition and interpretation of data, critically revised the manuscript for intellectual content, and approved the final version. JN is the guarantor of this work and, as such, accepts full responsibility for the work and/or the conduct of the study, had access to the data, and controlled the decision to publish. All authors contributed to the article and approved the submitted version.
